# Translation and Psychometric Properties of the Persian Version of the Older Adult Lifestyle Scale

**DOI:** 10.1002/hsr2.71785

**Published:** 2026-02-01

**Authors:** Fatemeh Mehriyan, Neda Ahmadzadeh Tori, Hamid Sharif‐Nia, Samaneh Pourhadi, Mina Galeshi, Mostafa maleki

**Affiliations:** ^1^ Student Research Committee Babol Iran; ^2^ Social Determinants of Health Research Center Babol University of Medical Sciences Babol Iran; ^3^ Psychosomatic Research Center Mazandaran University of Medical Sciences Sari Iran; ^4^ Department of Nursing, Amol Faculty of Nursing and Midwifery Mazandaran Univrsity of Medical Sciences Sari Iran; ^5^ Social Determinants of Health Research Center Babol University of Medical Sciences Babol Mazandaran Iran; ^6^ Clinical Research Development Unite of Rouhani Hospital Babol University of Medical Sciences Babol Iran; ^7^ Department of Public Health, School of Health Shahrekord University of Medical Sciences Shahrekord Iran

**Keywords:** life style, older adults, psychometric, qestionnaire, reliability, validity

## Abstract

**Background:**

The global increase in older adults necessitates reliable tools to assess lifestyle factors that influence healthy aging. Existing Western lifestyle assessment instruments often lack cultural relevance for non‐Western populations, including Iran. This study aimed to translate and assess the psychometric properties of the Persian version of the Older Adult Lifestyle Scale (P‐OALS) among community‐dwelling older adults in Iran.

**Methods:**

In this cross‐sectional methodological study, for construct validity assessment, a total of 397 community‐dwelling older adults aged 60 years and above were recruited from the city of Babol, Iran. Participants were selected using convenience sampling. The P‐OALS was translated using the Forward‐Backward method and rigorously evaluated for psychometric properties. The translation process followed WHO guidelines. Face and content validity were evaluated through a panel review conducted by 10 subject matter experts. Additionally, it was further assessed through interviews with a sample of 10 older adults. Construct validity was tested using exploratory factor analysis (EFA) and confirmatory factor analysis (CFA). Reliability was tested through internal consistency and test–retest stability, while convergent/discriminant validity was analyzed using composite reliability and average variance extracted. The questionnaires were administered by trained researchers through structured face‐to‐face sessions with participants.

**Results:**

Face validity showed that 90% of items were rated as clear. Expert panel evaluation confirmed acceptable content validity (CVI > 0.79; CVR > 0.62). EFA revealed a robust four‐factor structure—Quality of Relationships (6 items), Preventive Behaviors (5 items), Nutrition (4 items), and Physical Activity (4 items)—accounting for 55.4% of the total variance. CFA confirmed excellent model fit (comparative fit index = 0.923, root mean square error of approximation = 0.064, parsimony normed fit index = 0.712). The P‐OALS demonstrated strong reliability (*α* = 0.86, ICC = 0.859) and validity, with all subscales meeting psychometric benchmarks.

**Conclusion:**

The P‐OALS is a valid, reliable, and culturally adapted instrument for assessing the lifestyle of older persons residing in Babol, Iran. Its concise format and contextual relevance make it valuable for research and clinical practice. Future studies should explore its applicability in broader populations and longitudinal settings to further establish generalizability.

**Abbreviations**
AGFI: adjusted goodness of fit index
AMOS: analysis of moment structure
AVE: average variance extracted
CFA: confirmatory factor analysis
CFI: comparative fit index
CMIN/DF: minimum discrepancy function by degrees of freedom divided
COSMIN: Consensus‐Based Standards for the Selection of Health Status Measurement Instruments
CR: composite reliability
CVI: content validity index
CVR: content validity rate
DF: degree of freedom
EFA: exploratory factor analysis
FMI: functional independence measure
GFI: goodness of fit index
ICC: intraclass correlation coefficient
IFI: incremental fit index
IS: impact score
MLEFA: maximum likelihood exploratory factor analysis
NFI: normed fit index
NNFI: non‐normed fit index
P: P‐value
PASE: physical activity scale for the elderly
PNFI: Parsimonious normed fit index
P‐OALS: Persian version of Older Adult Lifestyle Scale
RMR: root mean square residual
RMSEA: root mean square error of approximation
SPSS: statistical package for the social sciences
χ2: chi‐square
χ2/df: ratio of chi square to degrees of freedom

## 1 Introduction

Aging represents an inevitable biological process marked by complex biological, psychological, and social transformations across the lifespan. Recent decades have witnessed remarkable advancements in medical and healthcare sectors, leading to increased life expectancy and a consequent global surge in older adult populations [1]. According to World Health Organization (WHO) projections, the population aged 65 and older will surpass 800 million by 2025 [2], with developing nations accounting for 70% of this demographic shift [3]. Global demographic projections indicate that the population aged 65 and older will grow substantially over the next three decades, increasing from 761 million individuals in 2021 to approximately 1.6 billion by 2050 [4]. Projections indicate that Iran's population aged 65 and older will grow significantly in the coming decades, exceeding 4 million (10% of the population) by 2035 and reaching over 19 million by 2050 [4]. The aging onset is different in each country. In Iran, aging begins at 60 years [5]. This rapid demographic transition presents significant economic, social, and public health challenges that demand urgent attention.

The expanding older adults cohort has been accompanied by a rising prevalence of age‐related health conditions, including diabetes, cardiovascular diseases, and mental health disorders [6]. While these conditions can substantially diminish the quality of life, growing evidence demonstrates that lifestyle modifications may effectively prevent disease progression and enhance overall well‐being [7]. Lifestyle encompasses a constellation of habitual behaviors, with healthy lifestyles specifically comprising health‐promoting activities such as balanced nutrition, regular physical activity, effective stress management, positive social engagement, and avoidance of harmful behaviors. These behavioral patterns emerge from dynamic interactions between individual life opportunities and stable variables, including ethnicity, age, socioeconomic status, and socialization experiences [8]. Consequently, personal choices and available opportunities fundamentally influence the development of health‐related habits and behaviors [9]. Proactive health strategies incorporating physical activity, nutritional awareness, and stress reduction techniques can significantly mitigate health risks in older populations [10].

By consciously adopting healthy lifestyle practices, individuals can actively influence their aging trajectory, fostering vitality, independence, and sustained well‐being during later life stages. The intricate relationship between aging processes and lifestyle choices underscores the transformative potential of preventive measures in determining quality of life among older persons. Given the well‐established connections between aging, chronic conditions, and lifestyle factors, a comprehensive assessment of older adults′ attitudes and behaviors toward lifestyle components provides healthcare professionals with valuable insights for evaluating living patterns and designing targeted interventions [11].

While Western societies have developed numerous lifestyle assessment instruments—including the Health‐Promoting Lifestyle Profile II (HPLP‐II) [12], Short Form Health Survey (SF‐36) [13], WHOQOL‐OLD [14], and Physical Activity Scale for the Elderly (PASE) [15]—these tools frequently prove culturally inappropriate for populations with distinct social characteristics. In Iran, where significant sociocultural differences exist, and many older adults have limited formal education, the implementation of complex assessment tools presents considerable challenges.

Although several Persian‐language questionnaires have been developed, including Eshaghi′s 46‐item Elderly Lifestyle Assessment [16] and Montazeri′s Healthy Lifestyle Questionnaire for the Elderly (HEL) [17], these instruments either lack comprehensive lifestyle coverage or prove excessively lengthy for practical use. Eshaghi′s tool includes many items, which may be tiring for older adults to complete, and it does not fully address areas like social life or mental well‐being [16]. Montazeri′s HEL, while well‐designed, focuses mostly on physical health and misses key lifestyle areas such as sleep, leisure, and emotional health [17]. These issues make both tools less suitable for quick use in clinics or community settings. This underscores the pressing need for culturally sensitive, easily comprehensible assessment tools tailored to Iran′s older adults. In contrast, the Older Adult Lifestyle Scale addresses these gaps by offering a shorter, culturally appropriate tool that covers multiple lifestyle domains designed specifically for the elderly population. The Chinese Older Adult Lifestyle Scale showed strong reliability and validity, and factor analysis supported its structure with good model fit [18].

Cultural context plays a pivotal role in shaping lifestyle behaviors and their assessment. The translation and validation of the Older Adult Lifestyle Scale within Iran′s sociocultural framework offers significant potential for identifying lifestyle‐associated factors among older adults. Although originally developed in Brazil, the Older Adult Lifestyle Scale includes universal lifestyle domains that are relevant across cultures. Its concise and multidimensional structure, combined with cultural adaptation and validation in the Iranian context, makes it more practical and culturally appropriate than existing lengthy or narrowly focused Iranian tools.

Presently, the psychometric properties of the P‐OALS remain unevaluated within Iranian populations. This validation study contributes to ongoing instrument refinement while supporting cross‐cultural applicability in geriatric research. This study aimed to translate and validate the P‐OALS to assess lifestyle in older Iranian adults. By developing a culturally adapted assessment tool, this research provides clinicians and researchers with an effective instrument for evaluating lifestyle patterns in Persian‐speaking older persons, ultimately facilitating the development of targeted, culturally appropriate interventions to promote healthy aging.

## 2 Methods

### 2.1 Study Design and Setting

This cross‐sectional methodological study was conducted in Babol, Iran, between February and March 2025. The research comprised two sequential phases [1]: translation of the healthy lifestyle scale and [2] psychometric evaluation of its Persian version.

### 2.2 Participants, Sample Size, and Data Collection

The target population consisted of community‐dwelling adults aged ≥ 60 years residing in Babol. Inclusion criteria required participants to meet the age threshold and provide voluntary informed consent. Exclusion criteria encompassed cognitive impairment, severe comorbid conditions, or unwillingness to participate.

Following MacCallum′s recommendations for psychometric studies [19], we established a minimum sample requirement of 200 cases. To accommodate separate samples for construct validation analyses, we recruited 397 participants through convenience sampling. This approach ensured adequate statistical power for both exploratory and confirmatory analyses.

The questionnaires were administered by trained researchers through structured face‐to‐face sessions, beginning with a comprehensive explanation of the study′s objectives to ensure informed participation. In light of the high illiteracy rate among the target population, the questionnaires were completed by trained interviewers via direct interviews with each participant. This supervised administration protocol, conducted under the direct oversight of the research team, was implemented to fulfill several methodological aims: to maintain a response rate exceeding 95%, to provide immediate clarification in response to participant inquiries, and to minimize the incidence of missing data through real‐time verification of responses. This approach contributed to enhanced data quality and ensured uniformity in the administration process across all participants.

### 2.3 Original Questionnaire

The study employed the Older Adult Lifestyle Scale, originally developed by Dr. Luana Ferreira at the Federal University of Juiz de Fora [8]. This instrument contains 19 items organized into four distinct subscales: preventive behaviors (5 items), nutrition (4 items), physical activity (4 items), and quality of social relationships (6 items). Each item utilizes a standardized 5‐point Likert‐type scale (1 = never to 5 = always), yielding potential total scores ranging from 19 to 95 points. In the original validation study by Ferreira et al. (2023), the scale demonstrated good internal consistency with Cronbach′s *α* values ranging from 0.74 to 0.88 across subscales. The scoring methodology involves a simple summation of all item responses, with higher aggregate scores reflecting more favorable lifestyle patterns.

### 2.4 Translation Procedure

Following formal authorization from the original scale developer, we implemented a rigorous Forward‐Backward translation protocol to ensure linguistic and conceptual equivalence. The questionnaire was sent to translators through email and later reviewed in in‐person sessions. The process commenced with two parallel translations performed by independent bilingual translators: one translator without specialized medical training (to preserve natural language usage) and one academic translator from a medical university (to maintain technical accuracy). These translations underwent systematic comparison and harmonization by a panel of three bilingual researchers to produce a consensus Persian version. An independent translator, blinded to the original English version, then performed a back‐translation of this preliminary Persian version into English. The back‐translated version underwent meticulous review by the original developer, Dr. Luana Ferreira, who provided expert feedback on semantic discrepancies and conceptual equivalencies. Through an iterative revision process incorporating this feedback, we achieved final approval of the Persian version.

### 2.5 Psychometric Evaluation

#### 2.5.1 Face Validity Assessment

The preliminary questionnaire underwent face validity testing through a dual evaluation process involving both target population representatives and subject matter experts. Ten older adults completed the instrument while providing detailed feedback on items of clarity, transparency, linguistic appropriateness, and comprehensibility of the items, which led to necessary modifications. The questionnaire was administered in face‐to‐face interviews by trained researchers. This ensured that participants with limited literacy could understand the items through verbal explanation. Among the 10 older adults in the face validity pretest, 90% rated the items as clear and comprehensible.

Then, an expert panel of 10 specialists in geriatrics, public health, and psychometrics conducted evaluations focusing on: the content, clarity, readability, simplicity, understandability of the questions, and practical administration considerations. All revisions were carefully documented and reviewed to ensure they maintained conceptual equivalence with the source questionnaire while optimizing cultural appropriateness for the Iranian older adults.

#### 2.5.2 Content Validity Assessment

To assess content validity, the Persian version of the questionnaire was reviewed by 10 subject matter experts. Content validity was evaluated both qualitatively (assessing wording, grammar, and relevance of items) and quantitatively by calculating the content validity ratio (CVR) and content validity index (CVI), as detailed below: To assess the questionnaire′s content validity, 10 experts in gerontology, health education, and psychometrics were recruited. They evaluated the instrument by completing it and providing feedback based on the CVI. For the CVR, each item was rated using three options: (a) *essential*, (b) *useful but not essential*, or (c) *not essential*. The CVR was calculated using Lawshe′s formula: *(Ne − N/2)/(N/2) *, where *Ne* = number of experts selecting “essential” and *N* = total experts. The cutoff value was determined using Lawshe′s table, which specifies a minimum CVR of 0.62 for 10 experts [20].

We used the CVI based on the relevance of each item, as recommended by Lynn [21]. A panel of experts rated each item on a 4‐point scale ranging from 1 (not relevant) to 4 (highly relevant), and CVI was calculated accordingly. The CVI score was calculated as the percentage of experts who rated an item as 3 or 4 (indicating high relevance). Items with a CVI > 0.79 were retained as highly appropriate, while those scoring 0.70–0.79 were revised for improvement. Items with a CVI < 0.70 were discarded for failing to meet the minimum validity threshold [22].

#### 2.5.3 Construct Validity

The factorial structure of the scale was examined through a two‐stage analytical approach. First, EFA was conducted using SPSS software to identify potential factor structures. This was followed by CFA in AMOS software to verify the emerging structure, with model fit indices evaluated to assess structural adequacy.

The sample size for EFA (*n* = 200) and CFA (*n* = 197) was determined based on established methodological guidelines for psychometric studies. For EFA, a minimum sample size of 200 is recommended to ensure stable factor solutions and reliable estimates, particularly when the communalities are moderate to high [23]. For CFA, a sample size of 197 aligns with the recommendation of at least 5–10 participants per estimated parameter [24], ensuring sufficient power to test the model fit. The first subset (*n* = 200) underwent Maximum Likelihood EFA with Promax rotation (Kaiser normalization) to explore the underlying factor structure. Data suitability for factor analysis was confirmed through the Kaiser–Meyer–Olkin measure (KMO > 0.8) and Bartlett′s Test of Sphericity (*p* < 0.01).

#### 2.5.4 Exploratory Factor Analysis (EFA)

A convenience sample of 200 eligible older adults from Babol completed both the Older Adult Lifestyle Scale and a demographic questionnaire assessing age, education level, and occupation. Sampling adequacy was verified through KMO (KMO = 0.70–0.80 [good]; 0.80–0.90 [excellent]) and Bartlett′s tests of Sphericity (*p* < 0.001) [25]. Maximum likelihood estimation with Promax rotation was employed for factor extraction. We applied the following retention criteria: minimum factor loading of 0.30, communality threshold of 0.20, and adherence to the three‐indicator rule requiring at least three items per factor [26]. The analysis determined the factor structure by calculating eigenvalues, which represent the variance in each item accounted for by the factor. The percentage of total variance explained by each factor was calculated by dividing the eigenvalue by the total number of items [27].

To ensure discriminant validity, we required items to demonstrate primary loadings ≥ 0.30 with cross‐loadings < 0.30 on secondary factors [28]. No items violated these thresholds.

#### 2.5.5 Confirmatory Factor Analysis (CFA)

A separate sample of 197 older adults completed the Older Adult Lifestyle Scale. Mardia′s multivariate skewness coefficient was 4.3. Model fit was assessed using multiple indices: incremental fit index (IFI > 0.90), Comparative Fit Index (CFI > 0.90), root mean square error of approximation (RMSEA < 0.05), Adjusted Goodness‐of‐fit InDex (AGFI > 0.80), Parsimony Comparative Fit Index (pcfi > 0.50), and Parsimony Normed Fit IndEx (PNFI > 0.5) [29].

#### 2.5.6 Normality, Outliers, and Missing Data

Univariate normality was evaluated using skewness ( ± 3) and kurtosis ( ± 8) thresholds. Multivariate outliers were identified via Mahalanobis *D*² (*p* < 0.001), while multivariate normality was assessed using Mardia′s coefficient of multivariate kurtosis [30]. Missing data per item ranged from 0.5% to 2.3%. To assess the impact of missingness, a sensitivity analysis using full information maximum likelihood (FIML) was conducted within the CFA. The results showed comparable model fit indices to those obtained from the original estimation, confirming the stability of the findings.

#### 2.5.7 Convergent and Discriminant Validity

The Older Adult Lifestyle Scale was assessed for both convergent and discriminant validity. Convergent validity was established using two criteria: composite reliability (CR > 0.7) and average variance extracted (AVE > 0.5) [31]. For discriminant validity, we employed the heterotrait‐monotrait ratio of correlations (HTMT) method, with all HTMT values required to be below 0.85 to confirm discriminant validity [32].

#### 2.5.8 Reliability Assessment

Internal consistency was evaluated using Cronbach's *α*, with values between 0.70 and 0.80 considered acceptable [33]. In addition to Cronbach′s *α*, McDonald′s *ω* was calculated as it provides more accurate estimates when factor loadings are not uniform. Given the ordinal nature of the Likert‐type items, ordinal alpha was also computed and showed similar reliability patterns. Stability was assessed through test–retest reliability in a subsample of 20 older adults who completed the P‐OALS twice at a 2‐week interval. The intraclass correlation coefficient (ICC > 0.7) was calculated to examine consistency between administrations [30]. Average inter‐item correlation (AIC: 0.2–0.4 is acceptable) was used to assess the internal consistency of a questionnaire [34].

#### 2.5.9 Feasibility and Acceptability

Feasibility was evaluated through completion time analysis and psychometric evaluation. The average completion time ranged from 10 to 15 min, demonstrating good practicality. Acceptability was assessed through participant feedback and questionnaire response patterns.

## 3 Data Analysis

Data were analyzed using SPSS v29 and AMOS v24. Analyses included descriptive statistics, EFA, CFA, reliability indices (α, ICC, Omega), and validity measures (AVE, CR, HTMT). CFA model fit indices were calculated using AMOS.

## 4 Results

### 4.1 Demographic Characteristics

The sample consisted of 397 older adults (mean age = 69.5 ± 8.6 years), with 62.5% (*n* = 248) men and 37.5% (*n* = 149) women. Most participants were married (75.25%, *n* = 301), while 24.75% (*n* = 99) were single. A majority (61.7%, *n* = 245) reported a history of chronic illness, and most had no formal education. Table 1 presents sociodemographic characteristics of the participants.

**Table 1:** Sociodemographic characteristics of the participants (*n* = 397).

Variable	Category	Number (%)
Gender	Male	248 (62.5)
	Female	149 (37.5)
Marital status	Married	313 (78.8)
	Single	84 (21.2)
Employment status	Retired	164 (41.3)
	Unemployed	197 (49.6)
	Employed	36 (9.1)
Type of employment	Governmental	156 (39.3)
	Self‐employed	241 (60.7)
Education level	Illiterate	164 (41.3)
	Primary	86 (21.7)
	Middle school	40 (10.1)
	High school	37 (9.3)
	University	70 (17.6)
Household income level	Low	136 (34.3)
	Medium	137 (34.5)
	Good	104 (26.2)
	Excellent	20 (5.0)
Chronic disease history	Yes	204 (51.4)
	No	193 (48.6)
Self‐rated health	Poor	25 (6.3)
	Good	327 (82.4)
	Excellent	45 (11.3)
Smoking/Alcohol history	Yes	63 (15.9)
	No	334 (84.1)

### 4.2 Face and Content Validity

Face validity showed that 90% of items were rated as clear. Expert panel evaluation confirmed acceptable face and content validity, with each item meeting the established thresholds (CVI > 0.79; CVR > 0.62).

### 4.3 EFA

**Figure 1:**
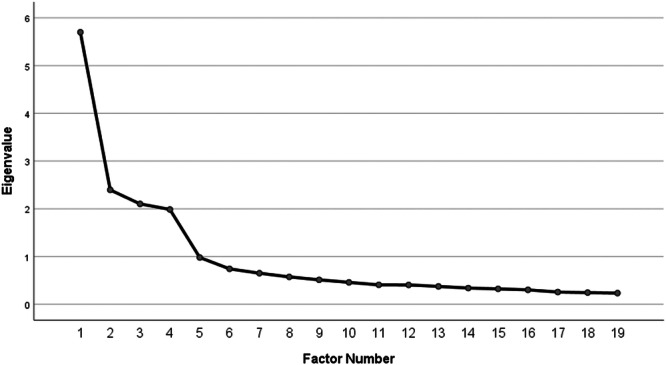
Scree plot of P‐OALS.

Maximum Likelihood EFA with Promax rotation revealed excellent sampling adequacy (KMO = 0.84) and a statistically significant Bartlett′s test of Sphericity (χ² = 2451.61, df = 190, *p* < 0.001), confirming that the correlation matrix was appropriate for factor analysis. The parallel analysis identified four factors accounting for 55.4% of the total variance (figure 1) [1]: Quality of Relationships (6 items, eigenvalue = 3.148) [2], Preventive Behaviors (5 items, eigenvalue = 2.785) [3], Nutrition (4 items, eigenvalue = 2.371), and Physical Activity (4 items, eigenvalue = 2.223) (Table 2). All items loaded cleanly on their hypothesized factors (range: 0.415–0.899) with no cross‐loadings ≥ 0.30, supporting unambiguous factor assignment. The inter‐factor correlations range from 0.274 to 0.341, indicating that while the factors are distinct, they demonstrate moderate correlations. This confirms that the factors were not truly orthogonal and justifies our use of oblique rotation (Promax).

**Table 2:** Factor structure of the Persian version of the older adults lifestyle scale: results of maximum likelihood exploratory factor analysis (*n* = 200).

Factors	Item	Factor loading	*h* ^*2*^	*ʎ*	Variance
Quality of relationships	**Q17.** Do you feel loved by your family members?	0.838	0.376	3.148	16.6
	**Q18.** Do you feel you have people you can trust?	0.803	0.370		
	**Q19.** Do you have people you can talk to?	0.766	0.665		
	**Q16.** Do you have a good relationship with the people you live with daily?	0.680	0.726		
	**Q14.** Do you have a good relationship with your family?	0.626	0.562		
	**Q15.** Do you feel supported by your friends?	0.570	0.445		
Preventive behaviors	**Q4.** Are you concerned about controlling your blood pressure?	0.899	0.721	2.785	14.7
	**Q3.** Are you concerned about controlling your blood sugar levels?	0.851	0.683		
	**Q5.** Do you visit the doctor regularly?	0.696	0.472		
	**Q2.** Do you perform preventive examinations such as mammograms, prostate exams, and others?	0.533	0.685		
	**Q1.** Do you care about preventing diseases such as diabetes, hypertension, obesity, and other illnesses?	0.488	0.611		
Food/diet	**Q7.** How often do you consume fresh foods such as rice, beans, fruits, vegetables, eggs, meat, milk, and others?	0.869	0.318	2.371	12.5
	**Q8.** Do you consume fruits and vegetables?	0.850	0.642		
	**Q9.** Do you consider your diet healthy?	0.654	0.477		
	**Q6.** Are you seeking to adopt a healthier and more nutritious diet?	0.460	0.422		
Physical activity	**Q10.** Do you engage in physical activity?	0.828	0.533	2.223	11.7
	**Q13.** Do you have the habit of walking at least 30 min a day?	0.819	0.673		
	**Q11.** Do you carry out daily activities such as gardening, housework, walking, and others requiring movement?	0.773	0.608		
	**Q12.** Are you concerned about maintaining regular physical activity?	0.415	0.542		

Abbreviations: h^2^, Communalities; λ, Eigenvalues.

### 4.4 Confirmatory Factor Analysis

**Figure 2:**
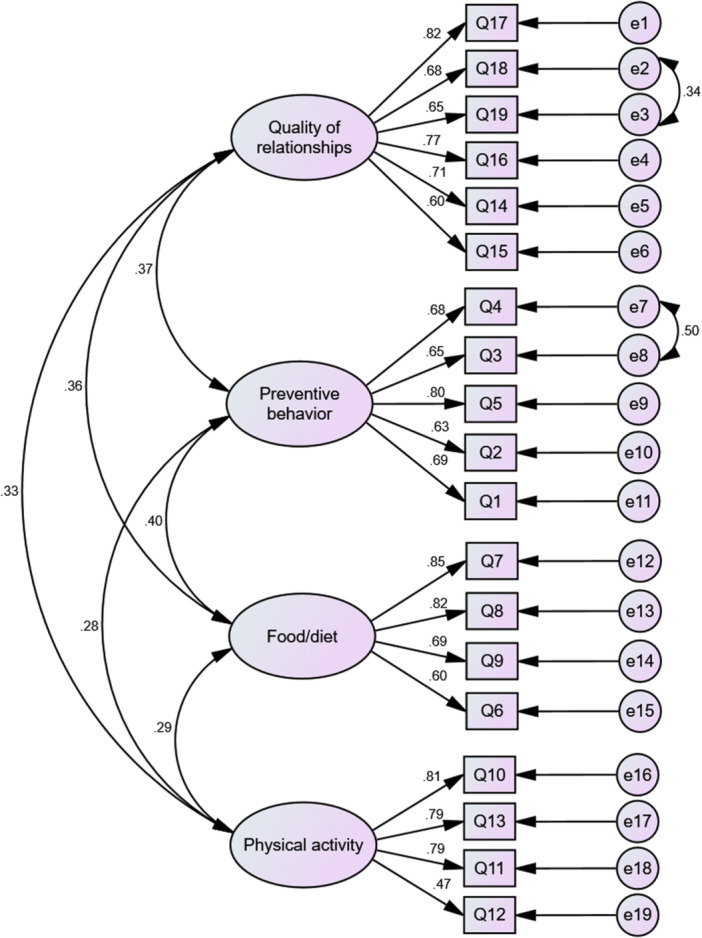
Confirmatory factor analysis of the Persian older adults lifestyle scale: standardized factor loadings and model structure (*n* = 197).

CFA was performed on the second subsample (*n* = 197) to validate the factor structure identified through MLEFA (Figure 2). The model demonstrated good fit: χ² [58] = 155.220, *p* < 0.001; *χ*²/df = 2.676; CFI = 0.923; NFI = 0.848; IFI = 0.925; TLI = 0.902; RMSEA = 0.064 (90% CI: 0.052–0.077). All fit indices met established thresholds for model acceptability (Table 3).

**Table 3:** CFA fit indices.

Fit Index	Value	Threshold	Status
CFI (comparative)	0.923	> 0.90	Good
IFI	0.925	> 0.90	Good
TLI	0.902	> 0.90	Good
RMSEA (absolute)	0.064	< 0.08	Acceptable
SRMR	0.048	< 0.08	Excellent
χ²/df	2.676	< 3.00	Acceptable
PCFI	0.731	> 0.50	Adequate
PNFI (parsimonious)	0.712	> 0.50	Adequate

### 4.5 Convergent and Discriminant Validity

Convergent validity was supported with CR > 0.70, and AVE > 0.50 for all constructs (Table 4). Discriminant validity was established using the heterotrait‐monotrait ratio. All HTMT values are < 0.85, supporting discriminant validity. More information is presented in Table 5.

**Table 4:** Convergent validity and construct reliability of the persian older adults lifestyle scale (*n* = 197).

Factor	AVE	CR	Ω (Omega)	α (Alpha)
Relationships	0.501	0.856	0.859	0.856
Preventive behaviors	0.481	0.821	0.822	0.814
Nutrition	0.553	0.830	0.815	0.810
Physical activity	0.534	0.815	0.808	0.802
Total scale	0.57	0.85	0.86	0.86

**Table 5:** Discriminant validity using HTMT ratio among constructs.

Constructs	Relationships	Preventive behaviors	Nutrition	Physical activity
Relationships		0.68	0.61	0.65
Preventive behaviors	0.68		0.71	0.73
Nutrition	0.61	0.71		0.66
Physical activity	0.65	0.73	0.66	

### 4.6 Reliability

All four factors demonstrated strong internal consistency (Cronbach′s α > 0.70), with the total scale showing excellent reliability (α = 0.86), McDonald′s ω > 0.70. Stability was confirmed by high test–retest reliability (ICC = 0.859, *p* < 0.001). AIC ranged from 0.20 to 0.40, indicating appropriate internal consistency.

## 5 Discussion

At present, few studies have systematically examined the lifestyle of older adults in Iran—a gap largely attributed to the lack of validated, culturally tailored instruments suitable for this population. The primary goal of the current study was to adapt and psychometrically validate the Older Adult Lifestyle Scale for older adults in Babol, Iran, thus creating the P‐OALS. This rigorous process incorporated best practices in cross‐cultural adaptation and psychometric validation, yielding a tool that is both reliable and culturally sensitive for use among Iranian older adults in Babol.

The demographic profile of the sample—predominantly women, married individuals, and with a substantial proportion reporting chronic illness—reflects trends commonly observed in gerontological research, where women are often overrepresented and multimorbidity is prevalent in later life [35, 36].

The psychometric evaluation of the P‐OALS demonstrated strong evidence of validity and reliability, aligning with best practices for instrument development in gerontology research. The high face validity (90% of items rated as clear) indicates that the scale′s wording and structure were well understood by older adults, a critical factor for ensuring accurate self‐reporting in populations with varied literacy and cognitive capacities [37]. Furthermore, the expert panel′s confirmation of acceptable content validity (CVI > 0.79; CVR > 0.62) mirrors the methodological rigor observed in the development of the original Older Adult Lifestyle Scale, ensuring that all items adequately represent the construct of lifestyle behaviors in later life.

The EFA identified a stable four‐factor structure—*Quality of Relationships*, *Preventive Behaviors*, *Nutrition*, and *Physical Activity*—accounting for 55.4% of the total variance, reflects the multidimensional nature of healthy aging as conceptualized in prior literature [35, 38, 39]. These domains parallel the final structure reported in Ferreira et al. [8], and corroborated by other cross‐cultural adaptations [40]. Importantly, all items loaded cleanly on their hypothesized factors with no problematic cross‐loadings, and moderate inter‐factor correlations supported the multidimensional yet interconnected conceptualization of lifestyle in older age [41].

One noteworthy observation was the relatively lower factor loading for item Q12 (“Are you concerned about maintaining regular physical activity?”) within the *Physical Activity* subscale (0.415). This may reflect cultural or contextual influences on how older Iranian adults interpret “concern” about physical activity. In collectivist societies such as Iran, health behaviors are often shaped by family and community norms rather than individual motivation, potentially diluting the emphasis on personal concern. Additionally, the phrasing of Q12 may have been perceived as more abstract compared to concrete behavior‐focused items like Q10 (“Do you engage in physical activity?”).

The CFA results further reinforced the factorial validity of the P‐OALS, with all indices meeting or exceeding conventional thresholds (CFI = 0.923; RMSEA = 0.064; PNFI = 0.712). These values are consistent with the original Brazilian validation [8] and with those reported in previous psychometric validations of lifestyle and health behavior instruments for older populations [40, 42]. The strong standardized factor loadings and adequate parsimony indicate that the P‐OALS effectively captures essential lifestyle dimensions in older adults without redundancy, thus maintaining brevity while ensuring comprehensive construct coverage and minimizing respondent burden.

Reliability analyses further reinforced the scale′s robustness. Cronbach′s α values for each subscale ranged from 0.80 to 0.85, and the total scale demonstrated excellent internal consistency (α = 0.86; ω > 0.80). The ICC of 0.859 confirmed strong test–retest stability, while item‐level analyses indicated that all 19 items contributed meaningfully to the construct. These results are consistent with psychometric benchmarks and the reliability indices reported for the original Older Adult Lifestyle Scale [8]. Convergent and discriminant validity were well established, with AVE values ≥ 0.50 and HTMT ratios < 0.85, confirming that each construct measures distinct yet related dimensions of lifestyle.

Among the extracted factors, *Quality of Relationships* emerged as the primary dimension, explaining the largest proportion of variance. This domain reflects the level of emotional and social support perceived by older adults through family and peer relationships. Evidence links high‐quality relationships to improved physical health outcomes, reduced stress, and greater cognitive resilience [38]. In Iran, where older adults are culturally respected and family bonds remain strong, the prominence of this factor underscores the sociocultural importance of interpersonal connectedness. Contrasting evidence from Schroeder et al. suggests that in some societies, technology‐mediated communication has supplanted in‐person contact as a key source of emotional support. In the Iranian context, however, traditional family structures and direct interaction remain dominant, potentially explaining the greater emphasis on tangible social ties. *Preventive Behaviors* reflects proactive engagement in health maintenance, aligning with literature on early detection and chronic disease prevention in older adults [43]. The *Nutrition* domain addresses dietary quality and consumption of fresh, nutrient‐dense foods, consistent with WHO and national dietary guidelines [44]. Finally, *Physical Activity* remains a cornerstone of functional independence, cognitive health, and reduced frailty risk [42].

The Chinese validation of the Older Adult Lifestyle Scale [18] closely mirrors the P‐OALS in structure, reliability, and validity, retaining the four original factors and demonstrating strong content validity (S‐CVI = 0.955). Reliability was high in both versions, though the Chinese scale showed exceptionally strong test–retest stability (ICC = 0.964 vs. 0.859) and explained more variance in EFA (76.68% vs. 55.4%). CFA fit indices were also slightly stronger in the Chinese study (CFI = 0.979, RMSEA = 0.053). In both contexts, “Quality of Relationships” emerged as a culturally significant domain, though shaped by distinct social norms—filial piety in China and traditional family bonds in Iran. These findings underscore the Older Adult Lifestyle Scale′s cross‐cultural robustness while highlighting subtle cultural differences in lifestyle interpretation.

Compared with the instruments currently used among Iranian older adults, such as the Activities of Daily Living (ADL) and Instrumental Activities of Daily Living (IADL) scales, the P‐OALS offers several important advantages. One of the most notable strengths is its cultural relevance. Because the scale was carefully translated and validated in Iran, it captures aspects of daily life that matter deeply in this context. While ADL and IADL measures remain useful for assessing independence, they focus narrowly on functional ability and do not provide a broader picture of lifestyle [45]. In contrast, P‐OALS takes a multidimensional approach, assessing relationships, preventive behaviors, nutrition, and physical activity, which together offer a more holistic understanding of healthy aging.

Another advantage lies in its psychometric strength. The validation process showed excellent reliability and validity, with indices such as CFI = 0.923, RMSEA = 0.064, and Cronbach′s *α* = 0.86, all meeting or exceeding accepted thresholds. This level of rigor is not always seen in imported tools used in Iran, which often lack thorough testing in local populations. Practicality is also a key benefit: unlike performance‐based ADL/IADL assessments that require trained raters and can be time‐consuming, P‐OALS is concise, self‐report, and easy to administer in both community and clinical settings [45]. Finally, by focusing on modifiable lifestyle factors, the scale provides actionable insights that can guide preventive health programs and tailored interventions—an especially valuable contribution given the high burden of chronic disease among older adults in Iran [46]. Taken together, these features highlight the added value of P‐OALS as a culturally relevant, psychometrically sound, and practically feasible tool for promoting healthy aging in Iran.

### 5.1 Limitations, Strengths, and Practical Implications

Nevertheless, some limitations should be acknowledged. First, the study was conducted in a single city (Babol) using convenience sampling, which may limit generalizability to the broader Iranian older adult population. Second, test–retest reliability was assessed in a small subsample (*n *= 20), which may reduce the robustness of stability estimates and should be viewed as a preliminary finding. Third, test‐retest validity was not evaluated due to the absence of comparable validated instruments. Furthermore, certain demographic variables—such as rural/urban residence, detailed socioeconomic indicators, and ethnicity—were not considered, though they may influence lifestyle patterns. Future studies should employ larger, randomized, and multi‐site samples and examine criterion validity alongside established lifestyle scales to strengthen external validity.

Despite these limitations, this study′s strengths include the rigorous cross‐cultural adaptation process, the use of both EFA and CFA for structural validation, and the application of multiple reliability measures including McDonald′s ω and Cronbach′s α.

From a practical perspective, the P‐OALS is highly feasible—it is brief (10–15 min to complete), accessible for older adults with low literacy, and generates actionable insights for healthcare providers. Its cultural adaptation supports applicability in Iranian geriatric research and practice, while its strong psychometric properties ensure consistent measurement across diverse demographics.

## 6 Conclusions

In conclusion, the P‐OALS demonstrated strong validity, reliability, and cultural relevance, making it a psychometrically sound tool for assessing the lifestyle of older adults in Babol, Iran. By filling a critical methodological gap, it offers researchers, clinicians, and policymakers a theoretically grounded and practically viable instrument for promoting healthy aging. Future research should explore its longitudinal performance, assess criterion validity, and test its applicability across diverse Iranian regions and cultural subgroups.

## Author Contributions

**Fatemeh Mehriyan** and **Samaneh Pourhadi:** Performance of data gathering. **Neda Ahmadzadeh Tori** and **Hamid Sharif‐Nia:** Planning and supervision of the work. **Hamid Sharif‐Nia** and **Mostafa Maleki:** Performance of the analysis. **Mina Galeshi**, and **Samaneh Pourhadi**, and all authors: Manuscript draft. **Neda Ahmadzadeh Tori**, **Mostafa Maleki**, and all authors: Comment on the final manuscript.

## Funding

The authors received no specific funding for this work.

## Ethics Statement

The Ethics Committee of Babol University of Medical Sciences (Babol, Iran) gave its approval to this study (Ethics code: IR. MUBABOL.HRI.REC.1403.298). The participants were given a thorough explanation of the study′s goals and methods, as well as assurances that their participation was entirely voluntary. Written Informed consent was obtained from all subjects. Permissions to use the data collection instruments were obtained from their developers. All procedures adhered to the appropriate guidelines and regulations.

## Consent

The authors and participants have given their consent for the publication of the study.

## Conflicts of Interest

The authors declare no conflicts of interest.

## Transparency Statement

The lead author Neda Ahmadzadeh Tori, Mostafa maleki affirms that this manuscript is an honest, accurate, and transparent account of the study being reported; that no important aspects of the study have been omitted; and that any discrepancies from the study as planned (and, if relevant, registered) have been explained.

## Data Availability

The data set used in this study will be available based on a reasoned request.
